# A study of community-acquired *Mycoplasma pneumoniae* in Yantai, China

**DOI:** 10.25100/cm.v49i2.3813

**Published:** 2018-06-30

**Authors:** Hong-Xia Yu, Mao-Mao Zhao, Zeng-Hui Pu, Yuan-Rong Ju, Yan Liu

**Affiliations:** 1 Pneumology Department, Shandong Provincial Hospital. Shandong University, Jinan, China; 2 Department of Infectious Diseases, Yantai Yuhuangding Hospital. Qingdao University, Yantai, China

**Keywords:** Mycoplasma pneumoniae, community-acquired pneumonia, adult, drug resistance, therapy, Mycoplasma pneumoniae, pneumonia, community-acquired, adultos, Resistencia a medicamentos, terapia

## Abstract

**Introduction:**

Community-acquired pneumonia (CAP) is a global disease responsible for a large number of deaths, with significant economic impact. As diagnostic tools have increased in sensitivity, understanding of the etiology of CAP has begun to change. *Mycoplasma pneumoniae* is one of the major pathogens causing CAP. Macrolides and related antibiotics are first-line treatments for *M. pneumoniae*. Macrolide resistance has been spreading for 15 years and now occurs in worldwide. We undertook the first study on macrolide resistance of *M. pneumoniae* in Yantai. This may be helpful to determine the appropriate therapy for CAP in this population.

**Objective:**

To investigate the rate and mechanism of macrolide resistance in Yantai.

**Methods:**

Pharyngeal swab samples were collected from adult CAP patients. Samples were assayed by polymerase chain reaction (PCR) and cultivated to test for *M. pneumoniae*. Nested PCR was used to specifically amplify *M. pneumoniae* 23S rRNA gene fragments containing mutations, and amplicons were analyzed by CE-SSCP for macrolide resistance mutations. Results were confirmed by sequencing. Twenty-seven strains of *M. pneumoniae* were isolated and the activities of nine antibiotics against *M. pneumoniae* were tested *in vitro*.

**Results:**

Out of 128 samples tested, 27 were positive for *M. pneumoniae*. Mycoplasma 100% macrolides resistance to *Mycoplasma pneumoniae*. The mechanism of macrolides resistance was A2063G point mutation in the sequence directly binding to macrolides in the 23S rRNA V domain *in vitro.* The mean pyretolytic time for the fluoroquinolone group was 4.7 ±2.9 d, which was significantly shorter than 8.2 ±4.1 d for the azithromycin group.

**Conclusions:**

Macrolides are not the first-line treatment for *M. pneumoniae* respiratory tract infections in Yantai.

## Resumen

**Introducción:**

Neumonía adquirida por en la comunidad (NAC) es una enfermedad responsable por un gran número de muertes y un impacto económico importante. Debido a que el diagnostico incrementó la sensibilidad, se cambió la etiología de la NAC. Adicionalmente, *Mycoplasma pneumoniae* es uno de los patógenos que causan la NAC. Los macrólidos y antibióticos relacionados son la primera línea de tratamiento para *M. pneumoniae.* La resistencia a macrólidos se aumentó en los últimos 15 años y ahora se encuentra distribuido en todo el mundo. Nosotros realizamos el primer estudio de resitencia a *M. pneumoniae* a los macrólidos en Yantai. Esto podría ser útil para determinar una terapia apropiada para NAC en esta población.

**Objetivo:**

Investigar la tasa y el mecanismo para la resitencia a los macrólidos en Yantai.

**Métodos:**

Se colectaron muestras faringeas usando un hisopo. Las muestras se analizaron mediante la reacción en cadena de la polimerasa (PCR) y por cultivo para *M. pneumoniae*. Se uso una PCR anidad para amplificar fragmentos del gen 23S rRNA especifico con las mutaciones para *M. pneumoniae*. Se analizaron amplicomes por CE-SSCP para determinar la resitencia a los macrólidos. Estos resultados se confirmaron por secuenciación. Se aislaron 27 cepas de *M. pneumoniae* y se probaron nueve antibióticos *in vitro.*

**Resultados:**

De 128 muestras, 27 fueron positivas para *M. pneumoniae.* Se determinó una resistencia a macrólidos por *Mycoplasma* del 100%. Los mecanismos de esta resitencia fue una mutacion punctual A2063G en la secuencia que se une directamente a los macrólidos en el dominio 23S rRNA V *in vitro.* El tiempo piotolítico medio para el grupo de fluoroquinolonas fue 4.7 ±2.9 d, que fue significativamente más corto que para el grupo de azitromicina: 8.2 ±4.1 d.

**Conclusiones:**

Los macrólidos no son la primera linea de tratamiento para las infecciones del tracto respiratorio contra *M. pneumoniae* respiratory tract infections en Yantai.

## Introduction

In recent years, researchers worldwide have identified *Mycoplasma pneumoniae* as one of the most common pathogens causing adult community-acquired pneumonia (CAP). In North America, Europe, Latin America, and Asia, *M. pneumoniae* pneumonia (MPP) accounts for 22%, 28%, 21%, and 20% of all pneumonia cases, respectively ^1^. In China, *M. pneumoniae* is responsible for 6.8%-38.9% of CAP etiology ^2^ ^-^ ^4^. Although MPP symptoms are mild in most adult patients, recent research shows that the incidence of severe MPP is increasing ^5^ ^-^ ^8^. Resistance of MPP to macrolide treatment is becoming a serious issue worldwide ^9^ ^-^ ^12^. Therefore, it is necessary to investigate drug resistance and drug resistance mechanisms of MPP, to determine the best treatment for CAP in the local community.

## Materials and Methods

### Inclusion criteria

Our study recruited CAP patients from the outpatient clinic and hospital admissions at Yantai Yuhuangding Hospital and its Laishan branch between January 2015 and January 2016. The inclusion criteria for this study were: 1) age >18 years; 2) meeting the diagnostic criteria for CAP issued by the American Thoracic Society and Infectious Diseases Society of America in 2007 ^13^; and 3) diagnosis of *M. pneumoniae* infection by positive pharyngeal swab polymerase chain reaction (PCR) test, according to the British Thoracic Society CAP guidelines ^14^. Patients were excluded if they: 1) presented with lung disease, such as lung abscess, aspiration pneumonia, or obstructive pneumonia; 2) were immunosuppressed, including AIDS patients, organ transplantation patients, patients taking two or more types of immunosuppressive drugs such as azathioprine, cyclosporine, and cyclophosphamide, or patients with malignant tumors who were actively receiving chemotherapy, radiotherapy, or surgical treatment; 3) were readmitted within 14 days from hospital discharge; 4) were pregnant or within 6 weeks after childbirth; 5) were previously enrolled in a similar study, were unable to provide medical history, or were unwilling to participate in this study.

### Patients

One hundred and thirty CAP patients were enrolled in this study. Two patients were lost to follow-up. Among the 128 cases finally enrolled, 27 were positive for *M. pneumoniae* (21.1%), including 11 males and 16 females aged between 18 and 72 years (average age 36 ±5.7).

### Mycoplasma pneumoniae isolation and identification

Patients’ clinical information was recorded. Pharyngeal swabs were obtained from each patient for *M. pneumoniae* identification and isolation. Pharyngeal swabs were either inoculated within 24 h after collection into 1 mL of CM403 medium (Oxoid, UK) with Mycoplasma Supplement G (Oxoid, UK) and incubated at 37° C, or preserved at -20° C. After 6 weeks, if the liquid medium changed from red to yellow, suggesting utilization of glucose, 0.2 mL of the culture was spread onto agar medium and incubated at 37° C with 5% CO_2_ for 7-14 days. A single colony was then isolated and subcultivated three times until “fried-egg” colonies, typical of *M. pneumoniae*, were observed. Identification of *M. pneumoniae* strains was performed using a molecular method. Genomic DNA was extracted from 2.0 mL of culture with single “fried-egg” colonies using the QIAamp DNA Mini Kit (QIAGEN, Germany), according to the manufacturer’s instructions. The *M. pneumoniae*-specific 16s rRNA gene fragment was amplified from the extracted DNA by PCR with the primer sequences used by Morozumi *et al* ^15^. The appearance of the target band on an electrophoresis gel indicated a positive *M. pneumoniae* infection.

### Antimicrobial susceptibility of M. pneumoniae

*In vitro* antimicrobial susceptibility tests were performed using erythromycin, azithromycin, moxifloxacin, levofloxacin, and tetracycline. The procedures and interpretation of results were conducted according to the “Methods for Antimicrobial Susceptibility Testing for Human Mycoplasma; Approved Guideline”^16^. *M. pneumoniae* reference strain FH (ATCC15531) was used as drug-sensitive control. Standard drugs were purchased from China Pharmaceutical Biological Products Analysis Institute.

### Detection of macrolide resistance-related genes

Domain V of the *M. pneumoniae* 23s rRNA gene was amplified by nested PCR (nPCR) of extracted DNA from clinical isolates, using previously described protocols and primer sequences ^15^. The nPCR products, including the reference strain, were sequenced (Sangon Biotech Co, Ltd., Shanghai, China). DNA sequences were compared to the *M. pneumoniae* strain FH sequence (GenBank accession no. CP002077.1) using BLAST.

### Statistical analysis

Data were analyzed by a t-test using SPSS 19.0 software. *p: <*0.05 suggested a statistically significant difference.

This study was conducted in accordance with the declaration of Helsinki. This study was conducted with approval from the Ethics Committee of Qingdao University. Written informed consent was obtained from all participants.

## Results

### Antimicrobial susceptibility

Eight of the 27 patients testing positive for *M. pneumoniae* used antimicrobial drugs 72 h prior to enrollment into the study. This included two patients using cephalosporin (7.4%), two patients using clindamycin (7.4%), three patients using macrolides (11.1%), and one patient using quinolones. All 27 strains (100%) of *M. pneumoniae* isolated from patients in this study were found to be resistant to macrolides. No quinolone- or tetracycline-resistant strains were identified (Table 1).

**Table 1:** *In vitro* drug sensitivity of the 27 isolated *M. pneumoniae* strains.

Strain No.	Erythromycin	Clarithromycin	Azithromycin	Tetracycline	Minocycline	Moxifloxacin	Gatifloxacin	Levofloxacin	Ciprofloxacin
1	128	128	64	0.50	0.250	0.064	0.064	0.50	1.0
2	64	64	16	0.25	0.125	0.032	0.064	0.25	0.5
3	128	128	64	0.50	0.500	0.125	0.125	0.50	1.0
4	≥256	≥256	128	0.25	0.125	0.064	0.064	0.50	0.5
5	64	128	32	0.50	0.250	0.064	0.064	0.50	0.5
6	64	64	32	0.50	0.250	0.064	0.064	0.50	0.5
7	128	128	32	0.50	0.250	0.064	0.064	0.50	1.0
8	128	128	64	0.50	0.500	0.064	0.064	0.50	0.5
9	128	128	64	0.50	0.250	0.064	0.064	0.50	0.5
10	64	64	32	0.25	0.250	0.032	0.064	0.25	0.5
11	≥256	≥256	128	0.25	0.125	0.064	0.064	0.50	0.5
12	128	128	32	0.50	0.250	0.064	0.064	0.50	0.5
13	≥256	≥256	128	0.25	0.125	0.064	0.064	0.50	0.5
14	128	128	64	0.50	0.250	0.064	0.064	0.50	0.5
15	≥256	≥256	64	0.25	0.125	0.064	0.064	0.50	0.5
16	128	128	64	0.25	0.125	0.064	0.064	0.50	0.5
17	≥256	≥256	128	0.25	0.125	0.064	0.064	0.50	0.5
18	≥256	≥256	64	0.25	0.125	0.064	0.064	0.50	0.5
19	128	128	64	0.50	0.250	0.064	0.064	0.50	0.5
20	≥256	≥256	64	0.25	0.125	0.064	0.064	0.50	0.5
21	≥256	≥256	64	0.25	0.125	0.064	0.064	0.50	0.5
22	128	128	64	0.25	0.125	0.064	0.064	0.50	0.5
23	128	128	64	0.50	0.250	0.064	0.064	0.50	1.0
24	128	256	16	0.50	0.250	0.064	0.064	0.50	0.5
25	256	256	64	0.50	0.125	0.064	0.064	0.50	1.0
26	128	128	64	0.50	0.250	0.064	0.064	0.50	1.0
27	128	128	64	0.50	0.250	0.064	0.064	0.50	0.5
Control	≤Control 64e	≤Control 64e	≤Control 64e	0.25	0.125	0.032	0.064	0.25	0.5

The MICs of erythromycin and clarithromycin (64-256 mg/L) were higher than that of azithromycin (16-128 mg/L). All 27 clinical isolates were susceptible to tetracyclines (tetracycline and minocycline) and fluoroquinolones (levofloxacin, ciprofloxacin, moxifloxacin, and gatifloxacin).

### 23S rRNA mutation in macrolide-resistant strains

We identified an A2063G point mutation in the sequence directly binding to macrolides in the 23S rRNA V domain of all 27 macrolide-resistant *M. pneumoniae* strains.

### Comparison between the clinical efficacy of azithromycin and fluoroquinolones

Among the 27 macrolide-resistant clinical strains, 9 cases were treated with azithromycin and 18 cases were treated with fluoroquinolones (10 cases with levofloxacin and 8 cases with moxifloxacin). The mean pyretolytic time for the fluoroquinolone group was 4.7 ±2.9 d, which is significantly shorter than 8.2±4.1 d for the azithromycin group (*p* <0.05). There was no statistical difference in the time for cough relief between the azithromycin group (13.5 ±7.4 d) and the fluoroquinolone group (8.6 ±5.1 d) (Table 2). 

**Table 2:** The effect comparison of the fluoroquinolones and macrolides groups.

Groups	Macrolides (N= 9)	Fluoroquinolones (N= 18)	*p* value
Total duration of fever (x̄±σ)	8.22 ±4.15	4.72 ±2.89	<0.05 (0.017)
Total duration of cough (x̄±σ)	13.56 ±7.37	8.67 ±5.05	>0.05 (0.053)

## Discussion

*M. pneumoniae* is the main cause of CAP in humans and is usually transmitted by close contact with infected patients. Studies have found that *M. pneumoniae* has replaced *Streptococcus pneumoniae* as the major CAP pathogen, accounting for 10%-30% of cases ^4^ ^,^ ^17^. Due to the lack of a cell wall in *M. pneumoniae* and fewer contraindications, macrolides have been the first choice for the clinical treatment of MPP ^18^ ^,^ ^19^.

Recently, more than 60% of macrolide-resistant *M. pneumoniae* strains in pediatric patients were shown to have high resistance to 14- and 15-membered ring macrolides. In contrast to pediatric patients, the prevalence of macrolide-resistant *M. pneumoniae* infection in adult patients in the United States and the UK is low at 0-23% ^20^ and 9.3% ^21^, respectively. However, several Chinese studies have reported a significantly higher percentage of macrolide-resistant *M. pneumoniae* strains, up to 90% ^22^.

In this study, we found that the prevalence of macrolide-resistant *M. pneumoniae* was 100% in Yantai, which is significantly higher than previous studies. This may be due to a lack of previous research into antimicrobial resistance status and the abuse of macrolides in local regions. As macrolides do not require a skin test and have few side effects, they have become commonly used antibiotics at home. Our study found that 37.5% (3/8) of patients had used macrolides before seeing a doctor. We also found that macrolide prescriptions were common in outpatient services of the Pediatric Department and the Respiratory Department in our hospital.

The mechanism of macrolide-resistance has been widely explored. Macrolide resistance in *M. pneumoniae* was found to be highly related to mutations in domain V of the 23S rRNA gene. The A2063G and A2064G mutations were identified as the most prevalent mutations related to macrolide resistance ^23^. The A2063G mutation, which often leads to high levels of macrolide resistance, can occur in Chinese populations at a frequency of more than 95% ^22^. In our study, 27 macrolide-resistant *M. pneumoniae* strains harbored the A2063G mutation in the 23S rRNA gene. This may be one of the reasons for 100% macrolide resistance in Yantai, China.

This study has several disadvantages. Firstly, the sample size was small. Secondly, due to concerns about macrolide resistance, MPP patients were mostly treated with fluoroquinolone and therefore, we were unable to analyze the effect of macrolide-resistance on prognosis. Thirdly, in this study we defined the diagnosis of MPP as isolation of *M. pneumoniae* in the pharyngeal swab. However, less than a quarter of patients had their diagnosis confirmed by paired serum antibody tests.

In summary, this study found that macrolide-resistant *M. pneumoniae* in adult CAP in Yantai is a major problem, with a resistance rate of 100%. Rigorous supervision of the administration of macrolides is urgently needed. Further studies on the genome of *M. pneumoniae* could provide us with information regarding drug resistance trends. At present, fluoroquinolone and tetracycline have good antimicrobial activity against *M. pneumoniae in vitro.* However, in Yantai, the abusive use of fluoroquinolones in fish and poultry industries could lead to the development of resistant *M. pneumoniae* strains. Management of antimicrobial drugs should be further strengthened, and drug resistance should be regularly monitored to avoid or delay the development of drug resistance.
